# Prevalence, Clinical Presentation Antifungal Susceptibility Profiles and Factors Associated with Chronic Pulmonary Aspergillosis among Pulmonary Tuberculosis Suspects in Uganda: A Cross-sectional study

**DOI:** 10.21203/rs.3.rs-11293911/v1

**Published:** 2026-10-08

**Authors:** Allen Rhoda Nankya, Richard Kwizera, Priscilla Apio, Moses Ssemwanga, Fiona Mutesi Magololo, Lorna Atikoro, Tonny Luggya, Amelia Margaret Namiiro, Moses Joloba, Willy Ssengoba, Beatrice Achan

**Affiliations:** 1 Department of Microbiology, Makerere University, Kampala, Uganda; 2 Infectious Diseases Institute, Kampala, Uganda; 3 Department of Immunology and Molecular Biology, College of Health Sciences, Makerere University, Kampala, Uganda; 4 College of Health Sciences, Makerere University, Kampala, Uganda

**Keywords:** Chronic Pulmonary Aspergillosis, Pulmonary Tuberculosis, Antifungal Susceptibility

## Abstract

**Background:**

Chronic pulmonary aspergillosis (CPA) is a possible diagnosis in patients evaluated for pulmonary tuberculosis (PTB), especially in resource limited settings with high TB burden. Its clinical presentation closely mimics PTB, often resulting in missed diagnosis and delayed initiation of treatment. This study aimed to describe the prevalence, clinical presentation, antifungal susceptibility profiles and factors associated with CPA among PTB suspects in Mulago National Referral Hospital.

**Methods:**

This was a cross-sectional study conducted between February and May 2025 at Mulago National Referral Hospital, Kampala, Uganda. We consecutively enrolled adult patients with persistent respiratory symptoms, suspected to have PTB. All participants underwent Gene Xpert MTB/RIF testing of sputum and fungal culture using Sabouraud dextrose agar. CPA was diagnosed based on the presence of compatible symptoms lasting two weeks or more, positive Aspergillus culture, and radiological findings. Antifungal susceptibility testing was done using gradient strips to determine minimum inhibitory concentrations. Multivariable logistic regression was used to identify predictors of CPA.

**Results:**

Of the 135 participants (median age 35 years, IQR: 28–49; 62.2% male), CPA was diagnosed in 13.3% (18/135), with 5.9% (8/135) having CPA with active PTB and 7.4% (10/135) having CPA without PTB. Additionally, 31.9% (43/135) had PTB without CPA, and 54.8% (74/135) had neither PTB nor CPA. The most common *Aspergillus* species isolated were *A. fumigatus* (38.1%), *A. niger* (38.1%), and *A. flavus* (28.6%). Common clinical presentations included productive cough, fever, hemoptysis, and chest pain in CPA cases. Pulmonary fibrosis (66%) was the most common radiological finding among CPA patients. Antifungal susceptibility testing revealed complete sensitivity to voriconazole while resistance to amphotericin B was observed in 31% of the isolates. Longer duration of symptoms (adjusted odds ratio [aOR]: 1.22 per week; 95% CI: 1.06–1.41; p=0.006), chest pain (aOR: 3.05; 95% CI: 1.02–9.96; p=0.052), and TB exposure (aOR: 3.57; 95% CI: 1.11–12.08; p=0.034) were independently associated with CPA,

**Conclusions:**

About 13.3% patients suspected to have PTB in Uganda have CPA with clinical presentation similar to PTB, mostly caused by *A. fumigatus* and *A. niger*. CPA should be screened among PTB suspects to improve early case detection and patient outcomes.

## BACKGROUND

Tuberculosis has become the leading cause of mortality by a single infectious disease surpassing COVID-19 disease after a 3 year dominance (1). Pulmonary tuberculosis (PTB) is a contagious respiratory infection caused by *Mycobacterium tuberculosis*, primarily targeting the lungs but capable of affecting other organs, leading to extrapulmonary TB (1,2). Despite being preventable and treatable, TB remains a global health challenge, an estimated 10.8 million people fell ill with TB worldwide in 2023 and over 80% of cases and deaths are in low- and middle-income countries (1). Sub-Saharan Africa (SSA) bears an uneven burden of TB, accounting for high morbidity and mortality rates due to socioeconomic disparities, inadequate healthcare access, and delayed diagnoses (3,4). Uganda, despite achieving nationwide TB treatment coverage, reported 12,000 TB-related deaths in 2022 (5). Dormant MTB infection, termed latent TB, affects one-quarter of the global population and carries a reactivation risk of 5–10%, especially among immunocompromised individuals (6).

Progression to active TB is influenced by risk factors such as chronic illnesses, malnutrition, poverty, and delayed healthcare access (7). PTB symptoms are nonspecific, including chronic cough, fever, weight loss, and night sweats (8). Diagnosis relies on chest imaging, which reveals diverse pulmonary abnormalities, including cavities, nodules, and fibrosis (9). Confirmatory diagnostic methods include Acid-Fast Bacilli (AFB) smear microscopy, culture on Lowenstein-Jensen media, and the GeneXpert MTB/RIF assay, all of which use sputum samples (10). Treatment involves a combination of first-line antibiotics: Rifampicin, Isoniazid, Pyrazinamide, and Ethambutol (11). Drug resistance necessitates the use of second-line regimens, which are more toxic and less accessible (12).

Chronic pulmonary aspergillosis (CPA) is an emerging concern, particularly among PTB patients, due to the immunosuppressive effects of TB, corticosteroid use, and long-term antibiotic therapy. CPA, caused predominantly by *Aspergillus fumigatus*, manifests with clinical features similar to PTB, including hemoptysis, chronic cough, and weight loss, often leading to misdiagnosis and delayed treatment (13,14). Aspergillus species are ubiquitous environmental molds, making inhalation of spores inevitable (15). CPA occurs when immunological or structural lung damage allows fungal colonization, often complicating pre-existing conditions like PTB, COPD, or sarcoidosis (16).

The recent CPA model using WHO’s global tuberculosis figures in 2020 estimated about 1.8 million cases arising yearly from previous PTB cases (14). With a population of about 45 million, it was estimated that CPA is responsible for 26,765 annual cases and 63,574 five-year-period prevalent cases of aspergillosis syndromes in Uganda (17). Early diagnosis and prompt antifungal therapy are critical for reducing mortality and improving outcomes (18). Conventional diagnostic methods for CPA include chest imaging, fungal culture, and serological tests such as galactomannan and Aspergillus-specific IgG detection (19). However, resource-limited settings often lack the capacity for these advanced diagnostics, resulting in high rates of misdiagnosis and poor patient outcomes (20). Antifungal resistance is a growing concern, driven by widespread antifungal use in agriculture, prolonged therapy in clinical settings, and suboptimal dosing practices (21). Azole-resistant Aspergillus species have been reported globally, particularly in regions with high antifungal usage (22,23). Understanding local antifungal susceptibility profiles is essential for guiding effective therapy and mitigating resistance.

The overlap in clinical presentations between PTB and CPA exacerbates diagnostic challenges, particularly in resource-limited settings. Over 42000 deaths occur within 12 months of misdiagnosis of pulmonary TB which lead to inappropriate treatments, prolonged hospitalizations, and economic loss (24,25). CPA management requires an integrated approach combining advanced diagnostics, antifungal therapy, and risk factor mitigation. This study addresses the critical knowledge gaps surrounding CPA in Uganda by investigating its prevalence, associated factors, clinical manifestations, and antifungal susceptibility profiles among PTB suspects.

Despite PTB being a major risk factor for CPA, and CPA being a major complication for PTB, there is limited data about the prevalence, clinical features, antifungal susceptibility profiles, and factors associated with CPA among Pulmonary TB suspects in Uganda. PTB suspects with CPA but having negative TB investigations are mostly given antibiotics they don’t need and sent back home without CPA investigations. Those with confirmed PTB and undiagnosed CPA at baseline often have poor prognosis and treatment outcomes.

The co-occurrence of CPA among PTB patients presents a significant and under recognized clinical challenge in Uganda. CPA often mimics PTB due to overlapping symptoms such as chronic cough, hemoptysis, and weight loss, leading to frequent misdiagnosis and delays in appropriate treatment (13,14). This diagnostic confusion is further exacerbated in resource-limited settings, where advanced diagnostic tools are scarce, resulting in inappropriate treatments, prolonged hospitalizations, increased healthcare costs, and higher mortality rates (20,24).

Uganda faces a high burden of TB, with 12,000 TB-related deaths reported in 2022 despite widespread treatment coverage (5). Additionally, CPA is estimated to cause 26,765 new cases and 63,574 five-year-period prevalent cases annually, primarily among individuals with a history of PTB (26). However, the prevalence and clinical characteristics of CPA remain underexplored due to limited research and diagnostic capacity. This lack of data contributes to the continued mismanagement of CPA, further worsening patient outcomes (18).

Emerging antifungal resistance, driven by widespread antifungal use in clinical settings, poses an additional challenge in CPA management (21,22). Azole-resistant Aspergillus species have been increasingly reported, complicating treatment and leading to poor clinical outcomes. Understanding local antifungal susceptibility profiles is crucial for guiding effective therapy, yet such data is scarce in Uganda (23).

Despite the significant morbidity and mortality associated with CPA, particularly in TB-endemic regions like Uganda, there is a critical gap in data regarding its prevalence, associated risk factors, and antifungal resistance patterns among TB suspects. This study seeks to address these gaps by investigating the prevalence, antifungal susceptibility profiles, clinical features and factors associated with CPA among Pulmonary TB suspects at a tertiary facility in Uganda. The findings will provide vital insights to improve diagnostic accuracy, inform clinical guidelines, and enhance patient outcomes in resource-limited settings. The findings aimed to justify why CPA point of care test should be added to the national TB programme at the point of TB screening to improve treatment outcomes for PTB suspects and patients and also improve antimicrobial stewardship.

The results from this study will justify why CPA point of care test should be added to the national TB programme at the point of TB screening to improve treatment outcomes for PTB suspects and patients and also improve antimicrobial stewardship. Findings from this study will directly inform clinical decision-making among physicians, enhancing the accuracy of CPA diagnosis and improving treatment strategies. This will contribute to reducing the rate of misdiagnosis between CPA and PTB, leading to better-targeted treatments and improved patient outcomes among presumptive PTB patients. Moreover, understanding the antifungal susceptibility profiles will support the selection of effective antifungal therapies, minimizing treatment failures and resistance development. Overall, the study’s findings are expected to yield better treatment outcomes, shorten hospital stays, lower healthcare costs, reduce misdiagnosis of CPA/PTB, and subsequently decrease morbidity and mortality rates among Pulmonary TB suspects.

## METHODS

### Study design and setting

The study employs a cross-sectional design to determine the prevalence, antifungal susceptibility profiles, clinical features, and associated factors of CPA among presumptive PTB patients. The research was conducted at Mulago Hospital’s TB Clinic, situated within the hospital’s premises in Kampala, Uganda. Additionally, to enhance the diagnostic capabilities and facilitate in-depth microbiological analyses, certain diagnostic procedures were conducted at the Makerere University College of Health Sciences at the Mycology Laboratory which is CAP accredited.

Mulago National Referral Hospital, situated in the Mulago Hill area approximately 4 kilometers north of Kampala’s central business district, stands as a prominent landmark and the largest hospital in Uganda. Nestled in the capital city of Kampala, Mulago Hospital serves as a vital hub for healthcare delivery, offering specialized medical services and serving as a referral center for patients nationwide. Within its premises lies the TB clinic, a crucial facility dedicated to the diagnosis, treatment, and management of tuberculosis. Equipped with state-of-the-art diagnostic facilities, including X-ray machines and laboratory equipment, the TB clinic plays a pivotal role in accurately diagnosing TB cases. Mulago Hospital’s TB Clinic and the mycology Lab were selected as study sites due to their specialized expertise, advanced diagnostic capabilities, and commitment to research.

### Participants

The target population for this study comprises individuals aged 18 years and older who present with clinical features indicative of pulmonary tuberculosis and are either seeking care or are referred to the TB Clinic at Mulago National Referral Hospital in Kampala, Uganda. These clinical features may include persistent cough lasting for two weeks or more among HIV negative patients but irrespective of the duration for HIV positive patients, often accompanied by hemoptysis, chest pain, significant weight loss, night sweats, fever, and fatigue.

Participants eligible for inclusion in the study were adults aged ≥18 years, individuals presumed to have pulmonary TB based on clinical features, and those who were able to provide informed consent. Individuals who were currently receiving or had received systemic antifungal therapy within the four weeks prior to enrollment were excluded. This criterion was necessary to avoid the suppression of fungal growth in culture, which could lead to an underestimation of the true prevalence of CPA, and to ensure that antifungal susceptibility testing results reflected the wild-type susceptibility profile of the isolates, unaffected by recent drug exposure. Individuals who were unable to provide sputum samples for mycological analysis were also excluded.

Chronic pulmonary aspergillosis (CPA) was defined based on clinical, radiological, and microbiological findings. Clinically, participants had respiratory and systemic symptoms consistent with CPA, including weight loss, persistent cough, and/or hemoptysis, while radiologically, chest images showed progressive cavitary infiltrates, a fungal ball, peri-cavitary fibrosis or infiltrates, and/or pleural thickening. Microbiologically, CPA was defined by a positive culture of *Aspergillus* species on Sabouraud dextrose agar.

Pulmonary tuberculosis (PTB) was defined based on clinical, radiological, and microbiological findings, with participants presenting with cough, fever, night sweats, weight loss, and/or hemoptysis, and chest images showing findings suggestive of pulmonary TB. Microbiologically, PTB was confirmed by sputum GeneXpert testing.

### Sample size calculation and sampling technique

The sample size was determined using the Kish and Leslie formula, (N = Z^2P(1-P)/D^2), where (N) was the total required sample size, (Z) was the Z-score corresponding to a 95% confidence level (1.96), (P) was the estimated prevalence of CPA among patients presenting with presumed pulmonary TB (9.7%), and (D) was the margin of error (0.05). Using these parameters, the calculated sample size was 134.6, which was rounded up to 135 participants. Therefore, 135 participants were recruited for the study.

### Study procedures

Socio-demographic information, clinical features (e.g., chronic cough, hemoptysis, and fatigue), prior TB treatment history, and radiological findings were collected through structured questionnaires and medical record reviews. Sputum samples were collected for mycological analysis. Chest X rays were done for all participants who had positive findings of fungal culture for aspergillus species.

### Specimen Collection, transportation and analysis procedures:

Early morning sputum samples were collected in sterile containers, with two samples obtained from each participant. The first sample was used for MTB GeneXpert testing to detect *Mycobacterium tuberculosis* DNA and assess rifampicin resistance. At the Mulago Hospital TB Clinic laboratory, the sputum was liquefied using sample reagent, transferred into GeneXpert cartridges, and loaded into the GeneXpert machine. The second sample was used for fungal culture, identification, and antifungal susceptibility testing and was transported at 2–8°C in courier boxes with ice packs to the Mycology Laboratory at Makerere University College of Health Sciences, where samples were stored at 4–8°C until processing.

Mycological analysis included direct microscopy, fungal culture, identification, and antifungal susceptibility testing. For direct microscopy, a portion of sputum was mixed with 10% potassium hydroxide (KOH), covered with a coverslip, allowed to digest for approximately 10 minutes, and examined under 10× and 40× objectives for fungal elements. For culture, sputum was streaked onto Sabouraud dextrose agar containing chloramphenicol and incubated at 25–30°C for 1–2 weeks, with daily monitoring for growth. Colonies were assessed based on morphology, texture, and pigmentation, while Lactophenol Cotton Blue staining was used for microscopic identification of characteristic fungal structures, including conidiophores and conidia. Antifungal susceptibility testing was performed using E-test gradient strips on RPMI 1640 agar supplemented with MOPS buffer and 2% glucose. Voriconazole and amphotericin B were tested, with plates incubated at 35°C for 48 hours, and minimum inhibitory concentrations (MICs) determined where the inhibition ellipse intersected the E-test scale.

Quality control was maintained throughout the study through adherence to standard operating procedures, training of research and laboratory personnel, routine monitoring and calibration of equipment, and regular quality assurance checks during data collection, specimen processing, and result interpretation. Collected data were also validated and checked for inconsistencies or errors before analysis to ensure the accuracy and reliability of the study findings.

### Statistical analysis

Categorical variables were summarized as frequencies and percentages, and continuous variables as medians and IQRs. Bivariate analyses used Chi-squared or Fisher’s Exact Tests for categorical variables and t-tests for continuous variables to compare CPA-positive and CPA-negative groups. The outcome variable, CPA diagnosis, was dichotomized (1 = CPA, 0 = No CPA). Variables with p < 0.2 in bivariate analysis or known risk factors (e.g. TB history) were considered for multivariable regression model. A backward stepwise logistic regression model was used, assessing interactions via two-way terms and likelihood ratio tests. Confounding was evaluated by a ≥10% change in odds ratios between models. Model fit was assessed with the Hosmer-Lemeshow test, and McFadden’s and Nagelkerke’s R^2^ were calculated. Crude and adjusted odds ratios (aORs) with 95% confidence intervals (CIs) were reported. A p-value < 0.05 was considered significant. Analyses were performed using R version 4.4.2.

### Ethical considerations

Ethical approval was sought from the Makerere University Department of Microbiology, School of Biomedical Sciences Research Ethics Committee (Mak-SBSREC) with approval number SBS-2024–648. Informed consent was obtained from all participants throughout the study. Administrative approval was sought from MNRH Ethics Committee to conduct this study at its TB clinic.

## Results

### 1. Baseline Characteristics of the study Participants

Of the 135 participants enrolled at Mulago National Referral Hospital, the majority were male (84; 62.2%), and the median age was 35 years (IQR: 28–49). All participants (100%) presented with a productive cough, consistent with inclusion criteria as pulmonary TB suspects. Gene Xpert was positive in 51 participants (37.8%), indicating active pulmonary TB.

Demographic and clinical characteristics were compared between CPA-positive (n=18) and CPA-negative (n=117) participants (Table 1). Fever was universal among CPA-positive participants (100.0%) compared to 90.6% in CPA-negative participants. Chest pain was more frequent in the CPA-positive group (61.1%) than in the CPA-negative group (41.0%). In contrast, night sweats were less common among CPA-positive participants (38.9%) compared to CPA-negative participants (50.4%). Other symptoms such as hemoptysis (50.0% vs. 47.9%), weight loss (44.4% vs. 41.9%), shortness of breath (27.8% vs. 38.5%), and wheezing (11.1% in both groups) showed similar frequencies between the groups. A longer duration of TB symptoms was observed among CPA-positive participants (median: 8 weeks, IQR: 4–11) compared to CPA-negative participants (median: 4 weeks, IQR: 3–8), with a statistically significant difference (p = 0.020). Exposure to TB was more common in CPA-positive participants (44.4%) than in CPA-negative participants (23.1%).

#### Prevalence of Chronic Pulmonary Aspergillosis

Overall, 18 of the 135 participants were diagnosed with CPA, yielding a prevalence of 13.33%. Among these, CPA was identified in both participants with and without active PTB. 29.6% (40/135) had PTB without CPA and 54.8% (74/135) had neither PTB nor CPA. The most commonly isolated Aspergillus species were *A. fumigatus* (8 participants, 5.9%), *A. niger* (8 participants, 5.9%), and *A. flavus* (6 participants, 4.4%), with less frequent species including *A. versicolor* (1 participant, 0.7%) and *A. terreus* (1 participant, 0.7%). Figure 1 illustrates the distribution of TB-Aspergillosis (5.9%), Aspergillosis without TB (7.4%), PTB without CPA (31.9%), and neither PTB nor CPA (54.8%).

#### Radiographic Findings among Participants with Positive Fungal Culture for *Aspergillus* species.

Chest X-rays results had varying patterns with the most common radiological feature being pulmonary fibrosis, seen in 12 participants. Cavitation alone was observed in 4 participants, and 2 had both pulmonary fibrosis and cavitation, 3 cases had normal chest X-rays. (Table 2)

#### Antifungal Susceptibility Findings of *Aspergillus* species isolated.

Amphotericin B susceptibility testing was done for 16 isolates 11 were sensitive, while 5 were resistant. Voriconazole susceptibility testing was performed for 8 isolates, all of which were sensitive to the drug. (Table 3)

#### Factors Associated with CPA

The duration of TB symptoms was independently associated with CPA (adjusted odds ratio [aOR]: 1.14 per week, 95% CI: 1.01–1.29, p=0.033), indicating that longer symptom duration increased the likelihood of CPA. Night sweats were inversely associated with CPA (aOR: 0.34, 95% CI: 0.12–0.97, p=0.044), suggesting their potential utility in distinguishing CPA from other post-TB conditions. Exposure to TB or fungal infections demonstrated a borderline significant association with CPA (aOR: 2.68, 95% CI: 0.96–7.49, p=0.060). Other variables-including gender, age, fever, weight loss, hemoptysis, shortness of breath, chest pain, wheezing, smoking history, alcohol use, and past medical history (asthma, bronchitis, COPD, post-TB lung disease, or previous TB treatment)-were not significantly associated with CPA in either bivariate or multivariable analyses (Table 4).

## Discussion

This study demonstrated that about 13.3% of patients presenting with presumptive TB at Mulago Hospital have CPA. The significant burden of CPA among PTB suspects in Uganda shows a critical diagnostic challenge in high-TB-burden settings, where CPA’s overlap with TB complicates management. The coexistence of CPA, particularly in patients with prolonged respiratory symptoms, suggests underdiagnoses in TB clinics, as symptoms are often attributed solely to TB. This overlap may lead to delayed or inappropriate treatment, especially in Gene Xpert-negative cases. For TB care, routine CPA screening in PTB suspects with chronic symptoms is essential to improve diagnostic accuracy. Clinically, integrating fungal diagnostics, such as Aspergillus culture, into TB algorithms could enhance outcomes. Policy-wise, national TB programs should recognize CPA as a comorbidity, allocating resources for fungal testing and addressing environmental and social risk factors, such as crowded living and poor ventilation, to reduce CPA and TB incidence.

The reported prevalence of CPA among individuals suspected of having TB varies significantly across studies, largely due to differences in diagnostic criteria and patient populations. According to a recent systematic review, approximately 9% of all patients undergoing TB treatment were found to have CPA, while the post-treatment prevalence was up to 13%. Notably, among those who continued to experience respiratory symptoms like in our study, the prevalence was 20% during treatment and as high as 48% following TB treatment, with a 95% CI of 36% to 61% (43). In Northern Uganda, CPA prevalence was 5% among post-TB patients (13), while in Nigeria, 8.7% of smear-negative PTB patients had CPA (44). Higher rates were reported in Gene Xpert-negative post TB patients in Indonesia (22%) (45) and post-TB patients in Vietnam (54.3%) (36). Our 15.6% prevalence among TB suspects aligns with these findings, particularly in settings where CPA may be misdiagnosed as smear-negative TB. Notably, 37.8% of our participants were Gene Xpert-positive, yet CPA was present in both Gene Xpert-positive and -negative groups, suggesting that CPA can mimic or coexist with TB in suspects. This supports the inclusion of CPA in the differential diagnosis for TB suspects, especially when microbiological confirmation is lacking.

Clinical features of CPA in TB suspects overlap significantly with TB, complicating diagnosis. All participants reported chronic productive cough, a hallmark of both conditions. However, hemoptysis was less frequent (48.1%) than reported in African CPA cohorts (up to 57%) in a review study by Olum and colleagues (46), where it contributes to mortality. Other symptoms usually termed “constitutional symptoms” such as fever, night sweats, and weight loss, were common but not significantly associated with CPA. This symptom profile suggests CPA may present subtly in TB suspects, necessitating high clinical suspicion. Persistent symptoms in TB suspects may also reflect Aspergillus-related conditions like Aspergillus bronchitis, particularly in those with post-TB lung damage (47)(48). This condition, characterized by positive sputum cultures without cavitary lesions, may account for some CPA cases in our cohort. Advanced imaging, unavailable in our study, is needed to distinguish these entities.

In our subset analysis of CPA-positive participants, antifungal susceptibility testing revealed complete sensitivity to Voriconazole, while resistance to Amphotericin B was observed in a notable proportion of cases. Radiological findings were heterogeneous, with pulmonary fibrosis as the most common abnormality, followed by cavitary lesions. These imaging patterns align with prior studies (19). These findings highlight the importance of integrating mycological testing into the diagnostic workup for TB suspects, particularly in high-burden settings where advanced imaging is limited.

Our study has several limitations. As a single-center study, findings may not represent all Ugandan TB suspects. Phenotypic identification of Aspergillus risked misidentification, and reliance on single sputum cultures increased contamination potential. Without serological testing or bronchoscopy, CPA detection was likely underestimated. Antibiotic susceptibility results have to be interpreted with caution as only few were reported and sample size was small limiting generalizability of findings. Future studies should address these gaps with multicenter designs, validated serology, and advanced diagnostics.

## Conclusions and recommendations

13.3% of patients evaluated for PTB in were found to have chronic pulmonary aspergillosis (CPA), with a substantial proportion co-infected with active PTB. The clinical overlap between CPA and PTB, particularly among individuals presenting with symptoms such as productive cough and chest pain, underscores the risk of misdiagnosis and delayed antifungal treatment. The dominant causative agents identified were *Aspergillus fumigatus* and *Aspergillus niger*, with pulmonary fibrosis being the most frequent radiological finding among CPA patients. Early CPA detection through targeted screening can improve case identification, optimize treatment strategies, and ultimately enhance patient outcomes in high TB burden settings.

### Recommendations

1. Integration of CPA Screening in TB Programs: CPA should be routinely screened for in all patients evaluated for PTB, especially those with persistent symptoms and negative Gene Xpert results, to reduce misdiagnosis and inappropriate TB treatment.
2. Adoption of Point-of-Care Testing: National TB control programs should consider incorporating CPA point-of-care diagnostic tools at the initial stage of TB evaluation to improve timely diagnosis and guide appropriate treatment.
3. Enhancement of Laboratory Capacity: Strengthening fungal diagnostic capabilities at national and regional referral hospitals, including routine fungal culture and susceptibility testing, is critical for guiding effective antifungal therapy and monitoring resistance patterns.
4. Clinical Training and Awareness: Clinicians and healthcare providers should be sensitized and trained to recognize CPA as a differential diagnosis in patients with chronic respiratory symptoms, particularly in TB-endemic settings.
5. Further Research: Larger prospective studies are needed to validate these findings, evaluate treatment outcomes, and assess the cost-effectiveness of integrating CPA diagnostics within national TB programs.

## Supplementary Material

Supplementary Files

This is a list of supplementary files associated with this preprint. Click to download.

- PrevalenceAntifungalSusceptibilityProfilesClinicalFeaturesandFactorsassociatedwithCPAamongPTBsuspectsatMNRHallversionslabels20250529163355

## Figures and Tables

**Figure 1:**
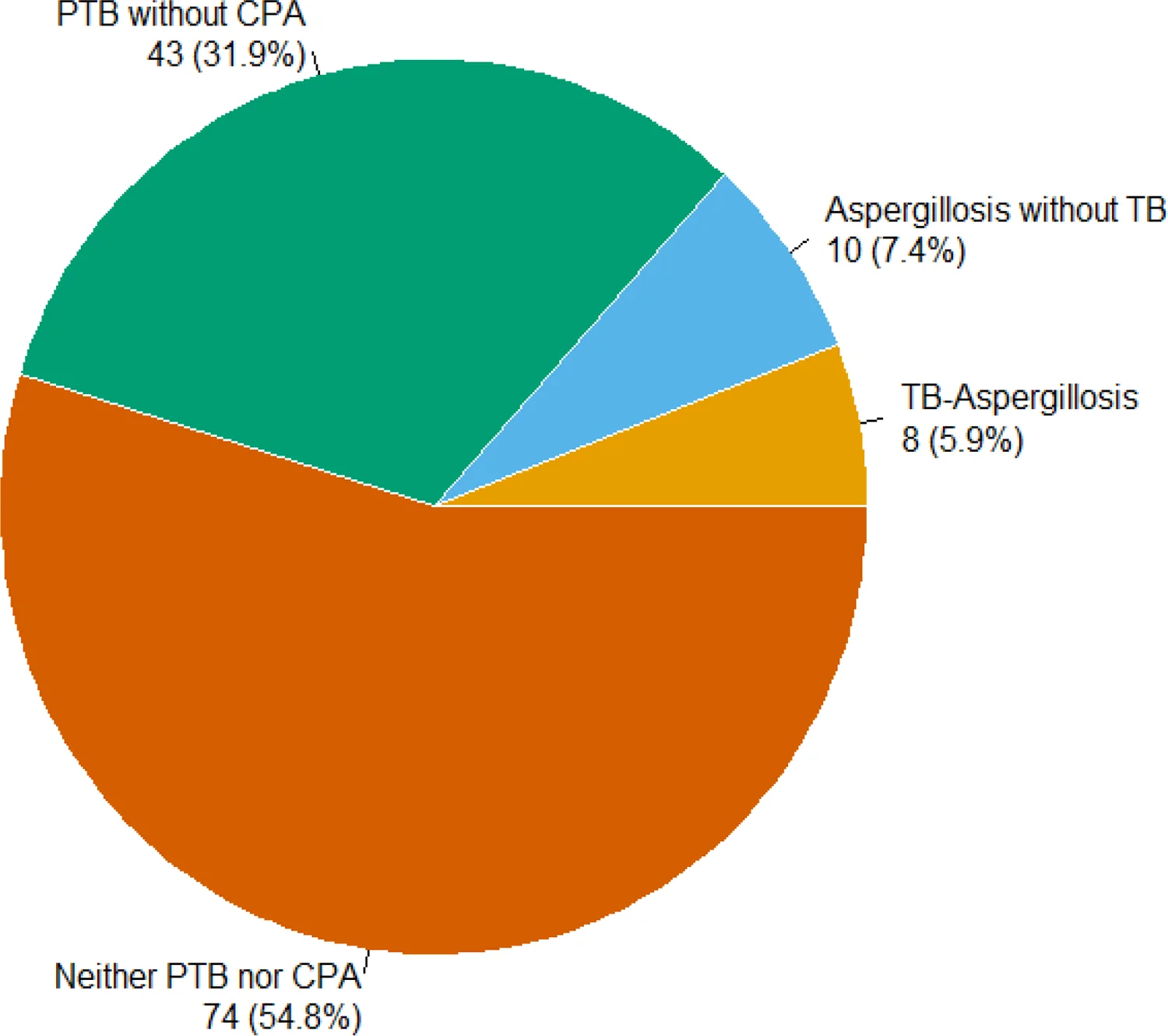
Distribution of CPA cases among Pulmonary TB suspects.

**Table 1.** Baseline Characteristics of study participants

Variable	Overall (n=135)	CPA Negative (n=117)	CPA Positive (n=18)	p-value
Age (years), median (IQR)	35 (28–49)	35 (28–49)	30.5 (26.2–50)	0.630
**Age Category**				0.662
<30	33 (28.2%)	33 (28.2%)	8 (44.4%)	
30–39	33 (28.2%)	33 (28.2%)	2 (11.1%)	
40–49	21 (17.9%)	21 (17.9%)	3 (16.7%)	
≥50	30 (25.6%)	30 (25.6%)	5 (27.8%)	
**Gender**				0.497
Female	51 (37.8%)	46 (39.3%)	5 (27.8%)	
Male	84 (62.2%)	71 (60.7%)	13 (72.2%)	
**Marital Status**				0.982
Single	49 (36.3%)	42 (35.9%)	7 (38.9%)	
Married	67 (49.6%)	58 (49.6%)	9 (50.0%)	
Divorced	9 (6.7%)	8 (6.8%)	1 (5.6%)	
Widowed	10 (7.4%)	9 (7.7%)	1 (5.6%)	
**Education Level**				0.582
No formal education	10 (7.4%)	9 (7.7%)	1 (5.6%)	
Primary	45 (33.3%)	41 (35.0%)	4 (22.2%)	
Secondary	61 (45.2%)	52 (44.4%)	9 (50.0%)	
Tertiary	19 (14.1%)	15 (12.8%)	4 (22.2%)	
**Occupation**				0.092
Employed	84 (62.2%)	73 (62.4%)	11 (61.1%)	
Student	18 (13.3%)	13 (11.1%)	5 (27.8%)	
Unemployed	33 (24.4%)	31 (26.5%)	2 (11.1%)	
**Income Level**				0.406
Below poverty line	30 (22.2%)	28 (23.9%)	2 (11.1%)	
Low income	87 (64.4%)	73 (62.4%)	14 (77.8%)	
Middle income	18 (13.3%)	16 (13.7%)	2 (11.1%)	
**Medical History**				
Tuberculosis History	26 (19.3%)	25 (21.4%)	1 (5.6%)	0.207
Other Respiratory Conditions	53 (39.3%)	44 (37.6%)	9 (50.0%)	0.457
Previous TB Treatment	18 (13.3%)	16 (13.7%)	2 (11.1%)	1.000
**Clinical Symptoms**				
Fever	124 (91.9%)	106 (90.6%)	18 (100.0%)	0.371
Night sweats	66 (48.9%)	59 (50.4%)	7 (38.9%)	0.510
Weight loss	57 (42.2%)	49 (41.9%)	8 (44.4%)	1.000
Hemoptysis	65 (48.1%)	56 (47.9%)	9 (50.0%)	1.000
Shortness of breath	50 (37.0%)	45 (38.5%)	5 (27.8%)	0.541
Chest pain	59 (43.7%)	48 (41.0%)	11 (61.1%)	0.179
Wheezing	15 (11.1%)	13 (11.1%)	2 (11.1%)	1.000
TB Symptom Duration (weeks), median (IQR)	4 (3–8)	4 (3–8)	8 (4–11)	**0.020**
**Risk Factors**				
Smoking History	58 (43.0%)	52 (44.4%)	6 (33.3%)	0.528
Alcohol Consumption	82 (60.7%)	72 (61.5%)	10 (55.6%)	0.822
TB Exposure	35 (25.9%)	27 (23.1%)	8 (44.4%)	0.102
Crowded Living Conditions	72 (53.3%)	61 (52.1%)	11 (61.1%)	0.648
**Residence**				0.987
Rural	64 (47.4%)	56 (47.9%)	8 (44.4%)	
Urban	71 (52.6%)	61 (52.1%)	10 (55.6%)	
**Laboratory Results**				
Gene Xpert Positive	51 (37.8%)	43 (36.8%)	8 (44.4%)	0.715
Gene Xpert Negative	80 (59.2%)	74 (0.63%)	10 (55.6%)	0.517

**Table 2.** Radiological Findings among Participants with Positive Fungal Culture for *Aspergillus* species.

Radiological Finding	Number of Participants (n)
Pulmonary Fibrosis only	12
Cavitation only	4
Pulmonary Fibrosis + Cavitation	2
Normal	3

**Table 3.** Antifungal Susceptibility Profiles of the *Aspergillus* species isolated.

Antifungal Agent	Susceptibility Status	Number of Participants (n)
**Voriconazole**	Sensitive	8
	Resistant	0
**Amphotericin B**	Sensitive	11
	Resistant	5

**Table 4.** Factors Associated with CPA

Characteristic	All n (%)	CPA Positive n=18 (%)	CPA Negative n=117 (%)	cOR (95% CI)	P-value	aOR (95% CI)	P-value
**Occupation**							
Employed	84 (62.2)	11 (61.1)	73 (62.4)	1.00 (ref)		1.00 (ref)	
Student	18 (13.3)	5 (27.8)	13 (11.1)	2.55 (0.80–8.15)	0.099	3.05 (0.75–12.27)	0.112
Unemployed	33 (24.4)	2 (11.1)	31 (26.5)	0.43 (0.09–2.00)	0.099	0.34 (0.05–1.57)	0.215
**Chest Pain**							
No	76 (56.3)	7 (38.9)	69 (59.0)	1.00 (ref)		1.00 (ref)	
Yes	59 (43.7)	11 (61.1)	48 (41.0)	2.26 (0.84–6.06)	0.179	3.05 (1.02–9.96)	0.052
**Exposure to TB**							
No	100 (74.1)	10 (55.6)	90 (76.9)	1.00 (ref)		1.00 (ref)	
Yes	35 (25.9)	8 (44.4)	27 (23.1)	2.67 (0.97–7.31)	0.102	3.57 (1.11–12.08)	**0.034**
**TB Symptom** **Duration** **(weeks)**							
Median – IQR	4 (3–8)	4 (3–8)	8 (4–11)	1.13 (1.01–1.27)	0.055	1.22 (1.06–1.41)	**0.006**

## Data Availability

The datasets used and/or analyzed during the current study are available.

## List of Abbreviations

AMR: Antimicrobial Resistance
CDC: Centers for Disease Control and Prevention
COPD: Chronic Obstructive Pulmonary Disease
CPA: Chronic Pulmonary Aspergillosis
DNA: Deoxyribonucleic Acid
DST: Drug Susceptibility Testing
HIV/AIDS: Human Immunodeficiency Virus/Acquired Immunodeficiency Syndrome
ICU: Intensive Care Unit
MOH: Ministry of Health
MTB: Mycobacterium Tuberculosis
PCR: Polymerase Chain Reaction
PTB: Pulmonary Tuberculosis
WHO: World Health Organization
